# Level and predictors of caring behaviours of critical care nurses

**DOI:** 10.1186/s12912-022-01125-4

**Published:** 2022-12-05

**Authors:** Fatma Refaat Ahmed, Ahmad Rajeh Saifan, Jacqueline Maria Dias, Muhammad Arsyad Subu, Rami Masadeh, Mohannad Eid AbuRuz

**Affiliations:** 1 Department of Nursing, College of Health Sciences, University of Sharjah, Sharjah, UAE; 2 Critical Care and Emergency Nursing Department, Faculty of Nursing, Alexandria University, Alexandria, Egypt; 3 Faculty of Nursing, Applied Science Private University, Amman, Jordan

**Keywords:** Caring behaviours, Critical care nurses, Jordan, United Arab Emirates

## Abstract

**Background:**

Advanced technologies in intensive care units, including artificial intelligence and digitization, has implications for psycho-emotional aspects of caring in terms of communication, involvement, and holistic provision in a safe, effective, and efficient manner. Critical care nurses must maintain a balance between their technological and humanistic caring behaviours during the provision of individualized holistic patient care. Therefore, this study was conducted to examine level and predictors of caring behaviours among critical care nurses in two Arab countries.

**Methods:**

A cross-sectional design was used to achieve the objective of this study, whereby a quantitative online questionnaire survey was administered to 210 adult intensive care unit nurses at two government hospitals in Sharjah (United Arab Emirates), and two university hospitals in Amman (Jordan). Based on G* Power analysis, 200 participants were adequate to run the analysis.

**Results:**

On average, 49% of the whole sample had ‘good’ caring behaviours. Among nurses who were working in Emirati intensive care units, 48.5% had good caring behaviours, compared to 47.4% of Jordanian intensive care unit nurses. Additionally, the results showed that predictors of caring behaviours among nurses include female gender, holding a master’s degree, interest in nursing profession, and a 1:1 nurse-to-patient ratio.

**Conclusions:**

About half of the ICU nurses in this study had low levels of caring behaviours. The present study highlights the requirement for integrating the concept of holistic and patient-centred care as the essence of the nursing profession in nursing curricula to improve the level of care provided by all nurses working in intensive care units. Continuing education programs and specific interventional programs should be directed toward predictors of caring behaviours among each specific group of nurses. Future research is needed using qualitative methods to understand what the perception of intensive care unit nurses is about caring.

## Background

Nurses are the largest professional group in the healthcare system, delivering care at all levels of the care continuum, and working around the clock caring for their patients [1]. Caring behaviours are feelings that provide a change in behaviour, safety and work according to standards. Caring behaviours are divided into two categories: instrumental behaviours, which are linked to technical and bodily activities; and expressive actions, which include psychosocial and emotional activities, such as providing patients with loyalty, confidence, hope, and emotional compassion [2]. It is expected that nurses’ caring behaviours are affected by a lack of confidence and experience, which is exacerbated in critical and high-pressure contexts such as the intensive care unit (ICU) environment, with complex patients’ health conditions, and diverse technologies [3, 4].

There are some factors that adopted these variations in caring behaviours among nurses working in different hospital units, especially in units with a high acuity, including workload, limited time, lack of commitment, job satisfaction, interest in the profession, and work environment [5, 6]. In addition to various characteristics of nurses, patients, and other stakeholders [7].

Previous research on caring behaviours mainly focused on the impact of the care delivery system, workplace climate, values, and cultural background [8, 9]. However, psycho-emotional aspects caring might be negatively affected with the continuous advancement in ICU technology, engagement of artificial intelligence, and increasing digitization in ICU care, for example, tele-ICU [10, 11]. ICU use of advanced technology has tended to tremendously limit chances to improve caring communication, involvement, and provision in a safe, effective, and time-saving manner, fundamentally undermining holistic and person-centred nursing care due to a skewed preoccupation in practice with technological (in addition to biomedical) aspects of care [12]. Therefore, critical care nurses should maintain a balance between their technological and humanistic caring behaviours during the provision of individualized holistic patient care [13]. The COVID-19 pandemic may also potentiate negative impacts on the way nurses cared for their critically ill patients [14].

Healthcare resources and diverse cultural identities are potential determinants and predictors of caring behaviours, thus it is necessary to study caring behaviours in various cultural and healthcare contexts. Recognizing predictors can help administrators and policy makers ensure quality of care more effectively. In the light of these changes and discrepancies, this study addresses the need for research exploring caring behaviours and its factors among critical care nurses in two Arab countries having similarities and differences in their prevailing cultural and health system characteristics. Few studies [6, 7] have determined correlation of predictors of caring behaviors, and there is no clear answer to the question of what factors predict nurses’ caring behaviours in ICUs. Therefore, this study was conducted in Jordanian and Emirati ICUs to explore the views of nurses working in different conditions. This was intended to facilitate identification of the similarities and differences between determinants of caring behaviours in two Arab countries, to answer the following research question:

- What are the most important predictors of nurses’ caring behaviours in ICUs in Jordan and UAE?

## Methods

### Design, sample, and settings

A cross-sectional design was used to achieve the objective of this study and to examine critical care nurses’ caring behaviours at a certain point in time. Participants were recruited conveniently from six adult ICUs of two governmental hospitals in Sharjah (UAE), and two university hospitals in Amman (Jordan), subject to the following inclusion criteria: (1) nurses who agreed to participate in the study, (2) nurses with a minimum of a bachelor’s degree, and (3) who had more than 1 year of active job experience in ICU. Nurses who were not proficient in the English language were excluded.

We used G* Power analysis to calculate the required sample size with the following assumptions: a medium effect size of 0.15, an α of 0.05, and a power of 0.8. In addition, multiple linear regression, with 13 independent variables, was used as a statistical test. Based on these assumptions, 131 ICU nurses were needed, thus 200 participants were adequate to run the analysis. However, to compensate for non-respondents and missing data, we recruited 226 ICU nurses, in which 210 were only respond (101 from UAE, and 109 from Jordan) with a high response rate of 93%.

### Instruments

A two-sectioned questionnaire was used to collect data: (1) demographic and work-related data, including gender, age, marital status, level of education, job position, working shift schedule (morning, evening, or night), nurse-patient ratio, and previous in-service education for caring behaviours; and (2) Caring behaviours Inventory 24 (CBI-24), adopted from Wu et al., [15], which included four subscales: ‘assurance’, ‘knowledge and skill’, ‘respectful’, and ‘connectedness’. The responses to the CBI items were on a 6-point Likert scale (from never to always). The overall internal consistency for both patients and nurses of CBI-24 is high (∝ = 0.96) according to Wu etal., [15], while in the current study it was found to be slightly lower (∝ = 0.93). The mean value was used for scoring caring behaviours in the four dimensions and at the overall level.

### Data collection procedure

Data collection was undertaken through an online survey administered using Google Forms and/or paper-based, according to participants’ preferences. First, ICU nurses mailing lists were obtained from the hospitals and an online survey form was created, and a link was sent to all participants via their emails after getting the approval of the selected hospitals. The survey form included detailed information about the study and consent of voluntarily participation. The average time to complete the questionnaire was 13 minutes. The authors were available physically or virtually for participants if any clarification was needed.

### Data analysis

Statistical Package for Social Sciences (SPSS Version 26.0) was used to run this analysis. Cronbach’s alpha was used to test the reliability of the tool. Descriptive statistics with frequencies and percentages or mean and standard deviation (SD) values were used to describe the demographic characteristics of the sample, in addition to the total score and subscale scores of the CBI-24. The two groups were compared with independent-sample t test, chi-square test and ANOVA /post hoc. For all used statistical tests, *P*-value equal or less than 0.05 was considered statistically significant. The research question of the study was tested by stepwise multiple linear regression.

### Ethical considerations

The study was approved by University of Sharjah Research Ethics Committee, UAE (Approval Reference No: REC-22-03-30-02-S), Applied Science Private University (IRB: Faculty 2021–2022-4 -5), Jordan, and the included hospitals (which remain anonymous). Informed consent was obtained from the participants, and they had the opportunity to either refuse participation or withdraw from the study at any stage. Anonymity and confidentiality were assured. All questionnaires were kept in a password-protected computer accessible only to the authors, and only aggregate data is used in this publication.

## Results

### Demographic characteristics

The participants were 118 (56.2%) women and 92 (43.8%) men with a mean age of 34 ± 7.2 years old. More than half (58.6%, *n* = 123) of respondents were married, and about two thirds of them (62.9%) had bachelor’s degree. The mean ICU work experience was 9 ± 6.07 years. More than three-quarters of the sample (77.1%) received communication education. The majority (85.7%, *n* = 180) of respondents had interest in the nursing profession, however, 48.1% of them reported that they intended to leave their organization. More than two thirds (72.4%) of the sample had a middle economic status. Approximately half of the respondents (51.9%) reported that they assigned to 2 patients per shift. Nurses who are working in Jordan had significantly longer work experience. However, they have significantly lower numbers in economic status compared with nurses who are working in UAE. Despite that the number of nurses who work in higher (nurse-to patient ratio) was significantly higher in Jordan compared to UAE, Jordanian nurses showed more interest in the nursing profession than those who are working in UAE. All other characteristics did not differ between the two groups (Table 1).

**Table 1:** Nurses’ characteristics based on country of practice (*N* = 210)

Items	Total sample	UAE (***n*** = 101)	Jordan (***n*** = 109)	t or χ2	***P***
**Age**	34 ± 7.2	33.9 ± 5.9	34.2 ± 8.2	−0.205	0.838
**Gender**					
Male	92 (43.8)	43 (42.6)	49 (44.9)	0.121	0.728
Female	118 (56.2)	58 (57.4)	60 (55.1)		
**Marital status**					
Single	65 (30.9)	32 (31.7)	33 (30.3)	11.27	0.010*
Married	123 (58.6)	53 (52.5)	70 (64.2)		
Divorced/ widowed	22 (10.5)	16 (15.8)	6 (5.5)		
**Education level**					
Bachelor	132 (62.9)	70 (69.3)	62 (56.9)	3.46	0.063
Master	78 (37.1)	31 (30.7)	47 (43.1)		
**Work experience** [years]	9.14 ± 6.07	6.6 ± 3.04	11.46 ± 7.2	−6.27	0.000*
**Prior communication education**					
Yes	162 (77.1)	78 (77.2)	84 (77.1)	0.001	0.56
No	48 (22.9)	23 (22.8)	25 (11.9)		
**Interest in nursing profession**					
Yes	180 (85.7)	82 (81.2)	98 (89.9)	3.26	0.04*
No	30 (14.3)	19 (18.8)	11 (10.1)		
**Turnover intention**					
Yes	101 (48.1)	44 (43.6)	57 (52.3)	1.44	0.15
No	109 (51.9)	57 (56.4)	52 (47.7)		
**Economic status**					
Low	41 (19.5)	26 (25.7)	15 (13.8)	9.99	0.007*
Middle	152 (72.4)	63 (62.4)	89 (81.7)		
High	17 (8.1)	12 (11.9)	5 (4.5)		
**Nurse-to-patient ratio**					
1:1	49 (23.3)	13 (12.9)	36 (33.0)	36.08	0.000*
1:2	109 (51.9)	45 (44.6)	64 (58.7)		
1:3	52 (24.8)	43 (42.5)	9 (8.3)		

Data are presented as n (%) or mean ± SD

*Abbreviation*s: *t* t-test, *χ2* Chi-square test, *P*: *P < .05

### Description of caring behaviours

There were statistically significant differences between the levels of all caring behaviours subscales (*P* = 0.002, 0.002, 0.000, respectively) among the two groups of nurses except the knowledge & skill subscale (*P* = 0.244). All of them, including the total score, revealed superior scores for the UAE participants (Table 2).

**Table 2:** Description of the individual items of the caring behaviours

Items	UAE	Jordan	t	***P***
**Assurance**	41.8 ± 4.07	39.3 ± 7.3	3.08	0.002*
**Knowledge & skill**	25.7 ± 2.7	25.1 ± 4.7	1.16	0.244
**Respectful**	30.4 ± 3.06	28.4 ± 5.6	3.14	0.002*
**Connectedness**	25.1 ± 2.86	22.8 ± 4.8	4.23	0.000*
**Total Score**	123.1 ± 8.9	115.6 ± 20.7	3.37	0.000*

*t* t-test, *P * *P < .05

There are no published mean norms for this tool [15]; therefore, researchers classified the caring behaviours score based on the median of their participants as a cutoff point. Consequently, nurses were classified as having high and low caring behaviours based on the median of the sample in this study, which was 121. Only 49% of the whole sample had good caring behaviours. Among nurses who were working in Emirati ICUs, 48.5% had good caring behaviours, compared to 47.4% of Jordanian ICU nurses.

### Comparison of caring behaviours

Of the whole sample, female nurses had significantly more caring behaviours compared to male nurses. Nurses with a master’s degree showed more caring to their patients than nurses with bachelor’s degree. Similar to the whole sample, female Jordanian nurses and those who were master holders had more caring behaviours than others did. In UAE, in addition to female gender and master’s degree, the less workload and higher economic status contributed to increasing nurses’ caring behaviours. UAE nurses working in the category of 1:3 was most responsible for the main effect difference in the total caring behaviours, having lower caring behaviours (120.6 ± 9.2) than those working in the category 1:2 (124.6 ± 8.6) and 1:1 (126.2 ± 7.6) (Table 3).

**Table 3:** Comparison of main study variables on categorical demographic variables

Variable	Category	Caring behaviours [Mean (SD)]					
		Total	t or F, P	UAE	t or F, P	Jordan	t or F, P
**Gender**	Male	115.2 ± 17.3	3.125	120.8 ± 7.02	−2.30	110.1 ± 21.9	−2.46
	Female	122.3 ± 15.3	0.002*	124.8 ± 9.8	0.023*	119.8 ± 18.9	0.015*
**Nurse-patient ratio**	1:1	117.5 ± 18.05	0.855	126.2 ± 7.6	3.18	114.3 ± 19.8	1.44
	1:2	118.8 ± 17.9	0.427	124.6 ± 8.6	0.046*	114.7 ± 21.4	0.242
	1:3	121.7 ± 11.3		120.6 ± 9.2		126.7 ± 18.4	
**Work shift schedule**	Morning	119.8 ± 16.3	1.019	124.2 ± 8.4	1.35	116.5 ± 19.8	1.36
	Evening	122.5 ± 12.1	0.363	124.2 ± 10.8	0.264	121.1 ± 13.4	0.259
	Night	116.8 ± 18.2		121.1 ± 9.3		109.2 ± 26.3	
**In-service communication skills training**	Yes	119.9 ± 15.03	−1.15	123.1 ± 8.6	0.094	117.0 ± 18.8	1.28
	No	116.8 ± 20.8	0.252	123.3 ± 10.02	0.925	110.9 ± 26.1	0.201
**Level of education**	Bachelor’s degree	121.3 ± 14.9	−2.39	121.4 ± 8.8	3.04	119.6 ± 20.1	2.76
	Master’s degree	115.7 ± 18.5	0.017*	127.06 ± 8.02	0.003*	108.7 ± 18.8	0.007*
**Economic status**	Low	114.9 ± 18.6	2.52	118.03 ± 6.6	6.74	109.5 ± 29.4	1.84
	Middle	119.8 ± 16.4	0.083	125.3 ± 9.1	0.002*	115.8 ± 19.2	0.164
	High	124.9 ± 9.14		122.9 ± 8.2		129.8 ± 10.2	

*t* t-test, *F* ANOVA /post hoc, *P* *P < .05

### Predictors of caring behaviours

The predictors of caring behaviours across the whole sample and individually for the participants in UAE and Jordan were as follows:

- Being a female nurse

Across sample: 0.2 points (β = 0.200, *p* = 0.004).

UAE: 0.18 points (β = 0.18, *p* = 0.036).

Jordan: .016 point (β = 0.163, *p* = 0.018)

- Having a master’s degree

Across sample: 0.28 points (β = 0.28, *p* = 0.022).

UAE: 0.23 points (β = 0.23, *p* = 0.016).

Jordan: 0.28 points (β = 0.28, *p* = 0.004).

- Interest in nursing profession

Across sample: 0.16 point (β = 0.16, *p* = 0.033).

Jordan: 0.21 points (β = 0.210, *p* = 0.034).

In addition, for UAE nurses working with 1:1 nurse patients’ ratio, this was an independent predictor which increased the total caring behaviours by 0.22 (β = 0.22, *p* = .025). The models explained 7, 16, and 15.3% of the variance, respectively, as shown in Table 4.

**Table 4:** Stepwise regression analyses for predictors of caring behaviours scores

**The whole sample (*N*** **= 210)**				
**Predictors**	**Standardized β**	***t***	***P***	**Model statistics**
Age	0.240	2.02	0.045	F (11,195) =2.51, *P* = 0.006 R^2^ = 7%
Female gender	0.200	2.88	0.004	
Interest of nursing profession	0.159	2.15	0.033	
Level of education: master holder	0.275	2.31	0.022	
**UAE (*n*** **= 109)**				
Female gender	0.178	1.68	0.036	F (11,89) =2.78, *P* = 0.004 R^2^ = 16%
Marital status: being married	−0.193	−1.83	0.017	
Level of education: master holder	0.227	1.89	0.016	
Nurse-patient ratio: 1:1	0.215	2.277	0.025	
**Jordan (*n*** **= 101)**				
Female gender	0.163	1.72	0.018	F (7,98) =3.72, *P* = 0.001 R^2^ = 15.3%
Level of education: master holder	0.277	2.91	0.004	
Interest in nursing profession	0.210	2.15	0.034	

In Table 4, variables entered in the whole sample model were age, marital status, working shift schedule, level of education, years of experience, previous in-service education for communication skills in block 1; and gender, interest in profession, intension to leave, economic status, and nurse-to-patient ratio in block 2.

Variables entered in UAE model were marital status, working shift, level of education, previous in-service education for communication skills, turnover intention, and economic status in block 1; and age, gender, years of experience, interest in profession, economic status, and nurse-to-patient ratio in block 2.

Variables entered in Jordan model were marital status, working shift schedule, level of education, previous in-service education for communication skills, turnover intention, and economic status in block 1; and age, gender, years of experience, interest in profession, economic status, and nurse-to-patient ratio in block 2.

## Discussion

The present study investigates caring behaviours among critical care nurses in ICUs in UAE and Jordan and evaluates caring behaviours predictors. In fact, nurses’ caring behaviours include technical and emotional activities that could directly impact patient’s safety and quality of delivered care [1, 2] . The main findings revealed that about half of the participants had low levels of caring behaviours, and discernible predictors that can increase caring behaviours among nurses include female gender, holding a master’s degree, interest in nursing profession, and 1:1 nurse-to-patient ratio.

### Description of caring behaviours

The proportion of nurses who had a high level of caring behaviours was 49%, which was a little bit decreased when we calculated it for each group separately. Recent studies reported that caring behaviours among ICU nurses are generally good [16, 17], and that overall caring behaviours are high when measured using the CBI-24 [18, 19]. This contradiction between studies [16–19] and our study might be attributable to differences in educational levels, attitudes, healthcare settings, workload, and even national (country) contextual factors, for example, culture barriers facing male nurses when caring for female patients. The full justification of this issue would require future international studies with large and diverse sampling from different Arab and other countries worldwide to explore how critical care nurses’ culture competency would affect their caring behaviours level. In addition, more clarification could be yielded using qualitative designs.

The findings also indicated that the subscales ‘assurance’ and ‘respectfulness’ had higher priority than ‘knowledge and skills’ and ‘connectedness’ in both Emirati and Jordanian ICUs. These findings indicated that participating critical care nurses pay more attention to provide psychological support through the assurance of human presence as well as professional and technical aspects of care, affirming earlier findings [19, 20].

### Comparison of caring behaviours

The present study revealed that female nurses demonstrated more caring behaviours among both groups of nurses while providing patient care, in alignment with previous studies [5, 21]. This would be related to the fact that females are kind-hearted, and also the conflict between masculinity and compassion/caring concepts could affect certain caring behaviours by male nurses. Our findings showed that nurses who were working in Emirati ICUs had less workload than those working in Jordanian ICUs, which could explain their higher caring behaviours levels. Numerous international studies have demonstrated that overloaded nurses are unable to provide appropriate or optimum care, including in terms of caring behaviours [5, 6, 22], which is particularly related to stress and burnout issues, which also drive high turnover in the healthcare professions [23, 24].

Consequently, these findings support a recommendation to lower nurse-to-patient ratios in order to increase nurses’ caring behaviours, and their ability to provide safe and high-quality care. However, this is inhibited by underlying worldwide structural challenges to the healthcare industry, including increasing demand and relatively shrinking supply of skilled professionals. As a short-term solution, soft skill programs can be offered to ameliorate stressful and demanding workplace conditions, including those that promote teamwork, in addition to behavioural and communication skills [23].

The present study also focused on comparing participants’ level of education. Nurses holding a master’s degree showed more caring attitudes toward their patients than their peers with bachelor’s degrees. This finding is worthy of further exploration. Studying more postgraduate courses may give nurses an opportunity for exposure to varieties of experiences and deeper understanding of the essence of caring. This finding is relatively novel and may be particular to the studied contexts. Educational level was not found to be a predictor of caring behaviours among nurses serving inpatient departments in a recent study aimed to assess the level and determinants of nurse caring behaviors [18], and several studies describing the educational level of their participants did not include this variable in their regression analysis [6, 7, 14].

We believe that the economic status would be a predictor for nurses’ caring behaviours, therefore, we included this variable in our study. However, our finding revealed insignificant difference between both groups of nurses. Only two recent studies were found that addressed this variable [5, 8].

Despite Jordanian nurses having more interest than those who are working in Emirati ICUs, and even having more work experience, the present study showed that nurses working in Emirati ICUs had a higher total caring behaviour score than those who were working in the Jordanian ICUs. This may be attributed to the lower nurse-to-patient ratio in Emirati ICUs, and the better economic status of nurses working in Emirati ICUs.

### Predictors of caring behaviours

Female gender was a mutual predictor of caring behaviours among UAE and Jordanian critical care nurses (i.e., across the whole sample). The study also showed that gender had also an influence on critical care nurses’ caring behaviours. This might suggest that educators should give more attention toward male nurses through emphasizing the value of caring [5]. Similar to gender, level of education was a common predictor between the two groups as well as the whole sample. This finding emphasizes the importance of the educational qualification, which allows opportunities for nurses to improve caring competencies, as reported in previous studies [25, 26].

Another step in the regression analysis indicated that interest in nursing profession was a significant predictor of caring behaviours after controlling other factors in the whole sample and among ICU Jordanian nurses, affirming a previous study which investigated priorities and predictors of caring behaviours in ICU nurses aiming at improving the quality of delivered care [7]. This finding could be due to lack of interest in nursing profession leading to a lack of professional caring, and thus worse outcomes for patients.

The findings of the present study also indicated that age across the whole sample was a significant predictor of caring behaviours, while it was not a predictor for Jordanian nurses and those who are working in Emirati ICUs alone, in contrast with a prior study which concluded that older nurses had less caring performance scores [5]. This might suggest that other factors could influence caring behaviours rather than age in each group independently.

Nurse-to-patient ratio was also a predictor for caring behaviours among nurses working in Emirati ICUs. Low nurse-to-patient ratio was associated with better caring for ICU patients, supporting previous findings [6, 27] which indicated that number of patients per shift is associated with caring behaviours scores. The regression analysis of nurses recruited from Emirati ICUs also revealed that married nurses were more caring toward their patients, contrary to other studies which indicated that married nurses had less caring behaviours sub-categories [5, 7].

### Conclusions and recommendations for practice

This study revealed that about half of the ICU nurses had low levels of caring behaviours. Female gender, holding a master’s degree, interest in nursing profession, and 1:1 nurse-to-patient ratio might be predictors of good caring behaviours among ICU nurses. More attention by nurse administrators should be directed toward men, and those with bachelor’s degrees (i.e., below postgraduate qualifications), as women and nurses with master’s degrees were found to exhibit better caring behaviours scores. It is recommended to integrate the concept of care as the essence of the nursing profession in nursing curricula. Future research is needed using qualitative methods to understand the perceptions of ICU nurses about particular aspects of caring in theory and practice.

### Strengths and limitations

This study has a lot of advantages that support its rigor, including the minimization of sampling bias due to a high response rate and comparatively large sample. This study helps further understanding of the predictors of caring behaviours in ICUs from the perspective of critical care nurses in low and medium-income Arab countries. However, a number of limitations must be acknowledged.

First, convenience sampling might make it harder to generalize our results. Second, using a solely quantitative approach can make it more difficult for the researchers to get full picture and obtain comprehensive data on participants’ caring behaviours. This is particularly problematic due to the subjective and complex nature of caring behaviours and holistic nursing care. Which is nevertheless crucial for clinical outcomes and service user satisfaction. Third, the use of the English version of CBI-24 might be considered a limitation. However, we did not use the Arabic one as it would be irrelevant to the majority of non-Arabic speaking expatriate nurses working in Emirati ICUs. Finally, it was challenging to compare the study results with prior studies due to the lack of published studies on nurses’ caring behaviours in ICUs specifically those that examining the impact of ICU technology/digitalization on quality of delivered care.

## Data Availability

All data generated or analysed during this study are available from the corresponding author [Fatma Refaat Ahmed] on request.

## Abbreviations

CBI-24: Caring behaviour Inventory-24
COVID-19: New Corona Virus
ICU: Intensive Care Unit
UAE: United Arab Emirates
